# An International Survey of Parental Attitudes to Technology Use by Their Autistic Children at Home

**DOI:** 10.1007/s10803-018-3798-0

**Published:** 2018-12-11

**Authors:** Margaret Holmes Laurie, Petra Warreyn, Blanca Villamía Uriarte, Charlotte Boonen, Sue Fletcher-Watson

**Affiliations:** 1 Patrick Wild Centre, University of Edinburgh, Edinburgh, UK; 2 Department of Experimental Clinical and Health Psychology, Ghent University, Ghent, Belgium; 3 Fundaciόn Orange, Madrid, Spain; 4 Patrick Wild Centre, Royal Edinburgh Hospital, Kennedy Tower, 23 Tipperlin Road, Edinburgh, EH10 5HF UK

**Keywords:** Autism spectrum disorder, Digital technology, Survey

## Abstract

Capturing variability in use of commercial technologies by autistic children can inform future learning and support technology design. Survey data were collected from parents (*n* = 388) in the UK, Spain, and Belgium, and includes information about individuals with a range of ages and ability levels. We found a comparable pattern of access and usage across age groups, though higher reading and language ability was linked to use of more devices and interfaces. Reported worries about technology correlated with longer time spent using technology. Autistic children use mainstream technologies for a broad range of recreational uses. The data suggest that technologies developed with therapeutic goals in mind may need to achieve a high standard of design to engage users.

## Introduction

Autistic people^1^ report high levels of use of technology—apps, software and online resources accessed through digital devices—for both leisure purposes and academic study (Hedges et al. 2018; Kuo et al. 2014; MacMullin et al. 2016; Mazurek and Wenstrup 2013). However less is known about the use of technology by autistic people who do not find a traditional survey design accessible—such as young children or those with learning disability. In the last decade, digital technologies with touchscreen, tangible or whole-body interfaces (e.g. the Nintendo Wii™) have become wide-spread, increasing accessibility of technology among these groups. Asking parents about how their children access these devices, and what functions they use them for, is a way to capture technology use in a diverse autistic sample which includes younger children and individuals with learning disability.

In addition, parent surveys can probe the inter-relation of technology use with parent attitudes. To date, research has largely focused on self-reporting by autistic adolescents and adults (Hedges et al. 2018; Mazurek and Wenstrup 2013), though one recent exploration of parent attitudes in a modest sample revealed enthusiasm but lack of knowledge among parents (Clark et al. 2015). It is likely that parent attitudes to technology both shape and are shaped by their child’s use of technology, as well as by external factors. For example, it has been reported that socioeconomic factors such as parent education and marital status predict what types of media are used by children in the home (Anand and Krosnick 2005) (but see Walker et al. 2011 who report no differences between parent demographics and use of technology in the home). Concerns felt by parents in general may be exacerbated for those with children who have a diagnosis of autism. Since autism is characterised by different social relationships, and sometimes isolation, parents may be worried that technology use detracts from or replaces “real life” interaction (Valkenburg and Peter 2009). The presence of restricted and repetitive behaviours may mean that the amount of time their child spends using technology is a particular source of anxiety for parents, as technology use itself may be considered ‘restricted and repetitive’ (Mazurek and Engelhardt 2013).

Systematic reviews indicate that digital technologies have the potential to deliver benefit in learning and developmental domains relevant to autism, such as communication (Ramdoss et al. 2011), social skills (Ramdoss et al. 2012), emotion recognition (Berggren et al. 2017) and academic skills (Pennington 2010). The quality of the evidence base is poor in most cases, however, with a recent attempt at a comprehensive meta-analysis showing that there are only a handful of well-controlled studies of technology use in an autistic sample (Grynszpan et al. 2014). Addressing this problem requires us to overcome a number of fundamental obstacles in the field, including fast-paced innovation in commercial technologies, which far exceeds the rate of output of academic research (Fletcher-Watson 2015). We also know that there is a systematic mis-match between those technologies for which independent studies of efficacy have been conducted, and those technologies which are available commercially (Kim et al. 2018; Ramdoss et al. 2012). On the whole, the technologies with the best quality evidence are often not the technologies that consumers can buy. This situation begs the question of what technologies autistic children and young people are actually using in the home. Knowing this is an important first step on the path towards building an evidence base for the use of technology to deliver benefit for autistic people.

Another open question in the field of technology for autism concerns the role of autism-specific design. There is a growing literature reporting on best practice in design with and for autistic users (Fletcher-Watson et al. 2016; Frauenberger et al. 2013), but as yet, little work directly connects design features with user experience or outcomes. Meanwhile, in the commercial sector, there is a substantial pool of apps for mobile touchscreen devices that are specifically marketed for users with autism (Fletcher-Watson and Durkin 2015), though a minority has any research evidence (Kim et al. 2018). It is not known whether autistic people really use these autism-specific technologies, or whether they are engaged equally—or more—with “off the shelf” mainstream technologies. In the design of technologies for autistic children, there has been much discussion about making pieces of technology accessible to users with motor and learning difficulties, and creating content which is appealing to a broad spectrum of users including those with a developmental delay (Fletcher-Watson et al. 2016), so it is interesting to explore if and how these are achieved by commercial technologies.

The current study aims to take a step towards addressing a series of gaps in our knowledge about autism and technology, while also providing some data on technology use since the popularisation of the iPad and other mobile touchscreen technologies. Our over-arching question is: how do children with autism use technology at home? By gathering parent report on the devices, interface types, software, and functions used by their children with autism, and estimates of time spent on technology-mediated activities, we can answer specific questions about use of technology by children with autism. First, is there evidence that children with autism frequently use autism-specific technologies, such as augmented and alternative communication devices and technologies designed specifically for autistic users? Second, how do parent attitudes to technology related to reported technology use by their child, and to demographics of the sample In order to increase generalizability of these data, we circulated the survey in three European countries (Belgium, Spain and UK) and included parents of autistic adults in the sample.

## Methods

### Participants

**Fig. 1:**
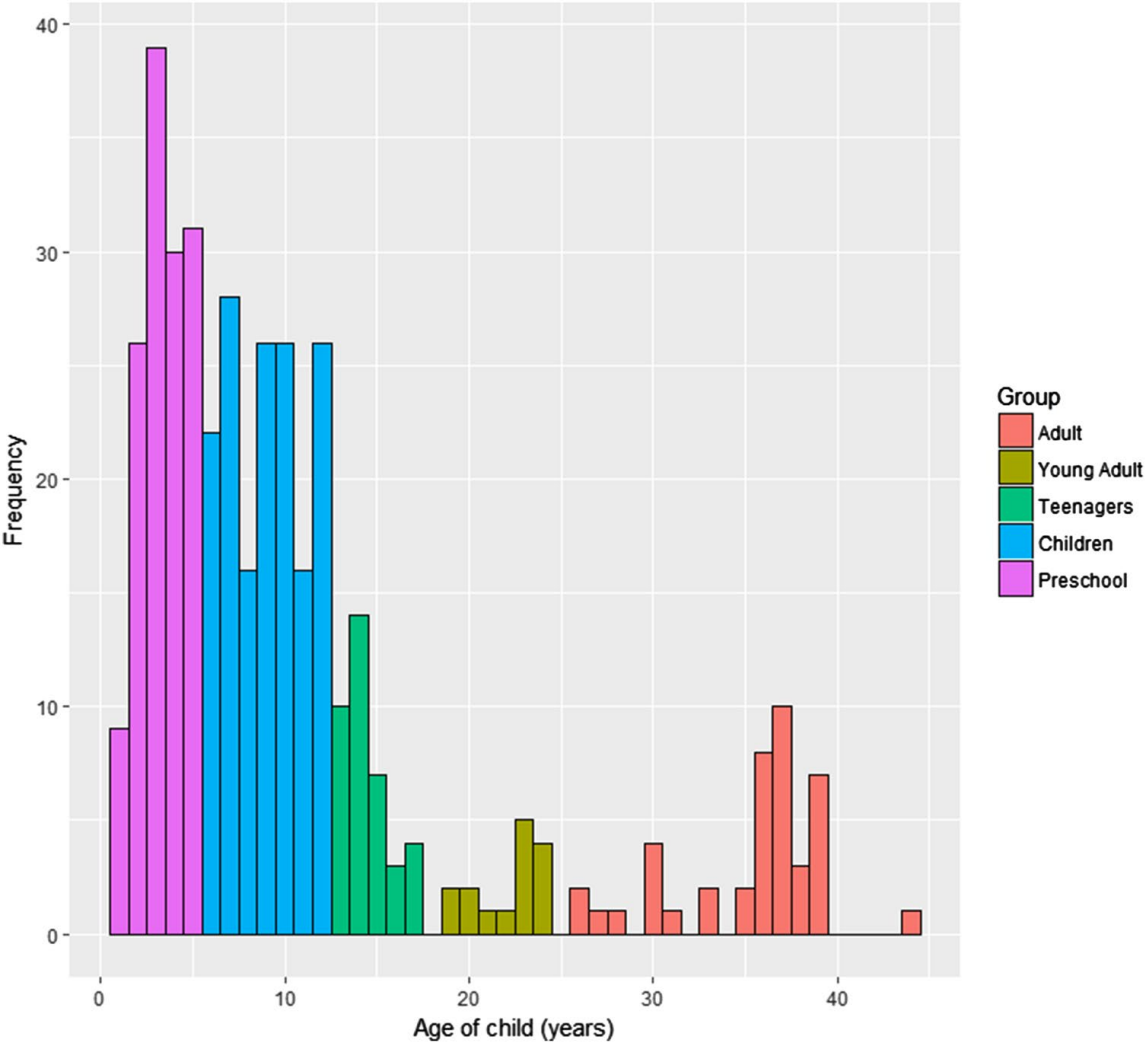
Distribution of represented children’s ages

Parents of children with autism were invited to complete the online survey in the UK, Belgium and Spain. We included data from children currently on a waiting list for diagnosis and those with an additional diagnosis such as Fragile-X Syndrome. The final sample (*n* = 388) contained 131 respondents from the UK, 134 from Spain and 123 from Belgium. Participants were split into five groups based on child’s age: “preschool children” aged 5 years and younger, “children” aged between 6 and 12 years, “teenagers” aged between 13 and 17 years, “young adults” aged between 18 and 25 years, and “adults” aged 26 years and older (Fig. 1).

Table 1 contains the samples sizes for data contributed broken down by both age group and country. Parents were also asked to report their own age, gender and most recent employment role (see Table 2), and their child’s age, diagnosis, together with an approximation of reading level, verbal language level, and some information about comorbidities and additional support needs (see Table 3).

**Table 1:** Distribution of data by groups of age and country

	Country			
Age group	UK	Spain	Belgium	Total
Young children (0–5 years)	29	42	63	134
Children (6–12 years)	72	62	26	160
Teenagers (13–17 years)	15	7	16	38
Young adults (18–25 years)	3	5	6	14
Adults (26+ years)	12	18	12	42
Total	131	134	123	388

**Table 2:** Parent demographics by groups of age and country

	Children (*n* = 332)			Adults (*n* = 56)		
	UK	Spain	Belgium	UK	Spain	Belgium
Age (median, IQR)						
	46 [41.75, 52.25]	44 [40, 48]	40 [36, 44]	54 [51, 59.5]	53 [49.5, 58.5]	49.5 [41, 52.75]
Gender						
Male	16 (13.79%)	28 (25.23%)	15 (14.29%)	2 (13.33%)	7 (30.43%)	3 (16.67%)
Female	98 (84.48%)	83 (74.77%)	81 (77.14%)	12 (80%)	15 (65.22%)	12 (66.67%)
Non-binary identification	1 (0.86%)	0	0	0	0	1 (5.56%)
Did not say	1 (0.86%)	0	9 (8.57%)	1 (6.67%)	1 (4.35%)	2 (11.11%)
Job role						
Professional	52 (44.83%)	61 (54.95%)	1 (0.95%)	6 (40%)	14 (60.87%)	1 (5.56%)
Non-manual skilled	21 (18.1%)	10 (9.01%)	1 (0.95%)	5 (33.33%)	1 (4.35%)	0
Manual skilled	5 (4.31%)	6 (5.41%)	12 (11.43%)	1 (6.67%)	0	2 (11.11%)
Partly skilled	3 (2.59%)	5 (4.5%)	38 (36.19%)	0	2 (8.7%)	7 (38.89%)
Unskilled	1 (0.86%)	4 (3.6%)	24 (22.86%)	0	1 (4.35%)	1 (5.56%)
Unemployed	0	1 (0.9%)	8 (7.62%)	0	2 (8.7%)	2 (11.11%)
Homemaker	26 (22.41%)	16 (14.41%)	0	0	3	(13.04%)
Full-time education	4 (3.45%)	3 (2.7%)	0			
Did not say	4 (3.45%)	5 (4.5%)	21 (20%)	1 (6.67%)	0	5 (27.78%)

**Table 3:** Child demographics by age and country

	Children (*n* = 332)			Adults (*n* = 56)		
	UK	Spain	Belgium	UK	Spain	Belgium
Age (median, IQR)						
	9 [5.75, 12]	7 [4, 9.5]	5 [3, 10]	37 [32, 37.5]	36 [26.5, 37]	30 [24, 35.75]
Gender						
Male	92 (79.31%)	82 (73.87%)	80 (76.19%)	10 (66.67%)	19 (82.61%)	14 (77.78%)
Female	17 (14.66%)	20 (18.02%)	8 (7.62%)	3 (20%)	2 (8.7%)	2 (11.11%)
Non-binary identification	2 (1.72%)	1 (0.9%)	0	0	0	0
Did not say	5 (4.31%)	8 (7.21%)	17 (16.19%)	2 (13.33%)	2 (8.7%)	2 (11.11%)
Learning disability						
Yes	68 (58.62%)	31 (27.93%)	47 (44.76%)	7 (46.67%)	8 (34.78%)	11 (61.11%)
No	41 (35.34%)	66 (59.64%)	40 (38.01%)	6 (40%)	12 (52.17%)	5 (27.78%)
Did not say	7 (6.03%)	14 (12.61%)	18 (17.14%)	2 (13.33%)	3 (13.04%)	2 (11.11%)
Reading ability						
Fluent	71 (61.21%)	39 (35.14%)	56 (53.33%)	5 (33.33%)	6 (26.09%)	13 (72.22%)
Learning	24 (20.69%)	31 (27.93%)	20 (19.05%)	4 (26.67%)	6 (26.09%)	1 (5.56%)
No	18 (15.52%)	31 (27.93%)	11 (10.48%)	4 (26.67%)	8 (34.78%)	2 (11.11%)
Did not say	3 (2.59%)	10 (9.01%)	18 (17.14%)	2 (13.33%)	3 (13.04%)	2 (11.11%)
Language ability						
Up to and including phrase speech	26 (22.41%)	50 (45.05%)	9 (8.57%)	7 (46.67%)	11 (47.83%)	2 (11.11%)
Including and beyond multi-part sentences	86 (74.14%)	46 (41.44%)	81 (77.14%)	6 (40%)	6 (34.78%)	14 (77.78%)
Did not say	4 (3.45%)	15 (13.51%)	15 (14.29%)	2 (13.33%)	4 (17.39%)	2 (11.11%)
Additional diagnoses						
Speech and language	5 (4.31%)	4 (3.6%)	10 (9.52%)	0	1 (4.35%)	1 (5.56%)
Attention Deficit Hyperactivity Disorder	13 (11.21%)	6 (5.41%)	15 (14.29%)	1 (6.67%)	3 (13.04%)	5 (27.78%)
Mental health	5 (4.31%)	0	6 (5.71%)	0	1 (4.35%)	2 (11.11%)
Dyspraxia	8 (6.9%)	0	2 (1.9%)	0	0	0
Physical disability	3 (2.59%)	0	0	0	1 (4.35%)	0
Sensory	5 (4.31%)	0	0	0	0	0
Multiple	8 (6.9%)	0	6 (5.71%)	1 (6.67%)	0	0
Developmental delay	0	8 (7.21%)	6 (5.71%)	0	2 (8.7%)	1 (5.56%)
Did not say	69 (59.48%)	93 (83.78%)	60 (57.14%)	13 (86.67%)	15 (65.22%)	9 (50%)

### Materials

The survey was developed in English, to collect parent demographics, child profiles, information about technology use at home, and attitudes towards technology use. To capture reading level, parents were asked “can your child read?” and selected from options “yes”, “learning to read” and “no.” To capture language level, parents selected the most complex level of language achieved by their child from a list of options: babbling, word approximations, single words, two-words together, short phrases, multi-part sentences, wh-questions, complex grammar, using pronouns appropriately and fluent, adult-like speech. Subsequently, translated versions were created by bilingual teams in Belgium and Spain. All versions were hosted by http://www.surveymonkey.com. A PDF copy of the survey in English, as well as data in an anonymised format, can be found at www.dart.ed.ac.uk/library under search terms ‘autism technology data.’

### Procedure

Ethical approval was granted from the Moray House School of Education Ethics Committee at the University of Edinburgh, and participants’ consent was inbuilt in the online survey. Parents of children with autism were invited to complete an online survey about their child’s technology use at home. The survey was advertised through a variety of social media outlets, including on the websites of charities (e.g. National Autistic Society, UK; Fundaciόn Orange, Spain), via the mailing lists of ASD-UK and DASLne (Database for children with autism living in the North East), and through the professional networks of the authors. The survey was online for approximately 2 months in each country. When the survey was closed, data were downloaded in .csv format and analysed using RStudio version 3.4.0 (RStudio Team 2015).

### Analysis Methods

Survey data were a mix of forced-choice responses (e.g. “select all the interface types that your child knows how to use”), 5-point rating scales (e.g. rate how strongly agree/disagree with the statement “I worry about how much time my child spends using technology” from strongly agree to strongly disagree) and free-text sections (e.g. “list the top five apps/softwares/online platforms used by your child”).

Data were split into five groups based on child’s age: “preschool children” aged 5 years and younger, “children” aged between 6 and 12 years, “teenagers” aged between 13 and 17 years, “young adults” aged between 18 and 25 years, and “adults” aged 26 years and older. Some data (*n* = 30) were excluded from these groups because the difference between parent age and child age was an invalid number (such as 0). In addition, we created sub-groups for analysis based on reading ability (fluent reader vs. learning vs. non-reader) and presence/absence of parent-reported learning disability. Verbal ability was condensed into two categories: the options “babbling, word approximations, single words, and two-words together” formed a “learning/delayed” group and the other options “short phrases, multi-part sentences, wh-questions, complex grammar, using pronouns appropriately and fluent, adult-like speech” formed a “fluent” group. When these are reported, we refer to these groups with the term “individual”, as these group allocations are independent of age (e.g. “individuals with/without learning disability”).

When comparing between groups (either by age or another demographic), we used Welch’s two-sample *t*-test (simple two group comparisons) and ANOVA (multi-group comparisons and interactions of within-group and between-group factors). Linear regression was used to probe the relations between technology use and demographic factors, and Chi-Square tests were used to check for multicollinearity. In all significance testing a *p*-value of < .05 is required for significance. In some cases—such as the analysis of the functions for which different technologies were used—we present data visually only and do not test for group differences. With regard to missing data, in each case, analysis is run on the data which are available on a case by case basis (including demographic information, information about technology access, etc.).

Parents in UK and Spain only were asked to name five specific apps, online platforms or softwares most used by their child using an open-response format. The most frequently mentioned apps were compiled into a “top ten” list. Note that, on occasion, apps were named as a group (e.g. “Toca Boca games” (sic)) and when this happened we summed responses naming specific apps in that category (e.g. Toca Tea Party, Toca Blocks) into a combined frequency count for all Toca Boca™ apps.

Parents reported the time per day that their child spent using technology for each device (e.g. time spent using a tablet, time spent using a games console) using a closed-ended set of time-windows. Data were converted to numerical values by taking the middle of the time window as an estimate of the time spent using the technology (e.g. 30–60 min was transposed to 45 min) and summed to create an estimate of total technology time per day. These scores were also used to calculate time spent using each kind of device, by age-group, in minutes per day. Note that in this case, each estimate draws on a different underlying sample, since different numbers of respondents had access to each device type.

The survey contained ten questions about parents’ thoughts about their child’s technology use. Responses were coded on a numerical scale from 1 to 5, and reverse coded when applicable so that high scores always reflect positive perceptions of technology. For ease of visualisation, the data were merged into three response categories (agree, neutral, disagree—collapsing “strongly” at each extreme) when plotted. An ‘attitude score’ was created from a subset of five relevant items from the questionnaire that measured attitudes to technology rather than (for example) available budget for buying new technologies. A Cronbach’s alpha threshold of 0.7 was set for scale reliability (Bland and Altman 1997). Two items were removed to achieve scale reliability (α = 0.62 on 5 items; α = 0.69 on 4 items; α = 0.76 on 3 items). The total attitude score was calculated from the three remaining items (“I worry about how much time my child spends using technology”, “I have had problems with my child being obsessed with technology”, and “Technology prevents my child from interacting with other people”) and a regression used to explore relationships between attitude score and participant demographics.

## Results

### Access to Device and Interface Types

Parents were asked which technology devices their child had access to in their home (Table 4) and which devices their child could use independently (Table 5). No specific time frame was indicated in the question regarding access to technology (the question was more about what devices are available in the home *at present*), but the question regarding time spent on technology was asked for any given day. For all age groups, the most reported devices which were available were tablets (iPad™ and other brands), and personal computers/laptops. The most popular games console was Nintendo Wii, closely followed by Nintendo DS™. Parents reported preschool children having access to median 4 devices (including gaming consoles) (interquartile range (IQR) = [2, 5], maximum = 10), children (up to 12 years) having access to median 5 devices (IQR = [3, 6], maximum = 10), teenagers (up to 17 years) having access to median 3 devices (IQR = [2, 5], maximum = 9), young adults (up to 25 years) having access to median 4 devices (IQR = [3.25, 5], maximum = 9), and adults (26 years and over) having access to median 3 devices (IQR = [1, 4], maximum = 8). The reported use of augmented and alternative communication devices (AAC) was very low in the sample—with only 5 parents reporting access in the home.

**Table 4:** Technology that children access in their homes

Device (%)	Preschool (*n* = 134)	Children (*n* = 160)	Teenagers (*n* = 38)	Young adults (*n* = 14)	Adults (*n* = 42)
iPad	68 (50.75%)	98 (61.25%)	18 (47.37%)	11 (78.57%)	18 (42.86%)
Tablet	54 (40.3%)	55 (34.48%)	9 (23.68%)	3 (21.43%)	14 (33.33%)
iPhone	34 (25.37%)	57 (35.63%)	13 (34.21%)	5 (35.71%)	8 (19.05%)
Smartphone	44 (32.84%)	61 (38.12%)	12 (31.58%)	5 (35.71%)	15 (35.71%)
Blackberry	1 (0.75%)	11 (6.88%)	2 (5.26%)	1 (7.14%)	0
PC	91 (67.91%)	119 (74.38%)	25 (62.79%)	11 (78.57%)	24 (57.14%)
Apple Mac	16 (11.94%)	28 (17.5%)	5 (13.16%)	5 (35.71%)	6 (14.29%)
Wii	52 (38.81%)	84 (52.5%)	16 (42.11%)	3 (21.43%)	12 (28.57%)
PlayStation	8 (5.97%)	44 (27.5%)	8 (21.05%)	9 (64.29%)	7 (16.67%)
Nintendo DS	51 (38.06%)	78 (48.75%)	14 (36.84%)	6 (42.86%)	12 (28.57%)
Xbox	17 (12.69%)	37 (23.12%)	7 (18.42%)	2 (14.29%)	6 (14.29%)
Kinect	8 (5.97%)	11 (6.88%)	3 (7.89%)	0	4 (9.52%)
AAC*	3 (2.24%)	1 (0.62%)	0	1 (7.14%)	0

*AAC augmented and alternative communication devices

**Table 5:** Interfaces which children can use independently

Interface (%)	Preschool (*n* = 134)	Children (*n* = 160)	Teenagers (*n* = 38)	Young adults (*n* = 14)	Adults (*n* = 42)
Large touchscreen	101 (75.37%)	124 (77.5%)	25 (65.79%)	11 (78.57%)	26 (61.9%)
Small touchscreen	95 (70.9%)	125 (78.12%)	26 (68.42%)	12 (85.71%)	27 (64.29%)
Mouse	97 (72.39%)	125 (78.12%)	29 (76.32%)	10 (71.43%)	18 (42.86%)
Keyboard	83 (61.94%)	119 (74.38%)	26 (68.42%)	11 (78.57%)	18 (42.86%)
Touchpad	76 (56.72%)	94 (58.75%)	20 (52.63%)	8 (57.14%)	15 (35.71%)
Joystick	66 (49.25%)	96 (60%)	17 (44.74%)	10 (71.43%)	9 (21.43%)
Wii	64 (47.76%)	91 (56.88%)	20 (52.63%)	7 (50%)	14 (33.33%)
Kinect	25 (18.66%)	36 (22.5%)	7 (18.42%)	3 (21.43%)	3 (7.14%)

Differences in the mean number of devices accessed by children were explored between age groups, learning disability (with vs. without), language ability (verbally fluent vs. delayed/learning) and reading level (fluent vs. learning vs. non-reader). An analysis of variance test (ANOVA) revealed that there was a statistically significant difference between the number of devices accessed by age groups; *F*(4, 383) = 4.167, *p* = .002). Post-hoc analysis using Tukey Honest Significant Difference (Tukey HSD) tests showed that significant differences were present between mean number of devices accessed between children and adults (*p* < .01), and children and pre-schoolers (*p* < .02), with all other comparisons showing non-statistically significant differences. There was no significant difference between mean number of devices accessed by individuals with a learning disability (mean = 4.39) and individuals without a learning disability (mean = 3.97; *t*(339.78 = − 1.89, *p* = .059). Verbally fluent individuals accessed more devices (mean = 4.55) than individuals with less verbal language production (mean = 3.31), and a statistically significant difference was reported; *t*(219.42) = − 5.59, *p* < .001. Finally, an ANOVA revealed a difference between individuals who were fluent readers (mean = 4.72), individualised who were learning to read (mean = 3.91) and individuals who could not read (mean = 2.75; *F*(2, 347) = 30.51, *p* < .001). A post-hoc Tukey HSD test showed that fluent readers had access to significantly more debices than those who were learning/could not read (*p* < .001), and those learning to read had access to significantly more devices than those who could not read (*p* < .001).

Parents were asked about which technology interfaces their child could use independently (Table 5). For nearly all interfaces, except large touchscreens, a higher percentage of pre-schoolers, children, and teenagers were able to use it independently, compared with adults. In each group, touch screen interfaces, followed by mouse and keyboard, were reported as more frequently independently used by individuals. The median number of interfaces that preschool children could use independently was 5 (out of 8 listed in the survey) (IQR = [2, 7]; maximum = 8), for children the median was 5.5 (IQR = [4, 7], maximum = 8), and for teenagers the median was 5 (IQR = [3, 7]; maximum = 8). The median number of interfaces that young adults could independently use was 5 (IQR = [3.25, 7]; maximum = 8), and for adults (aged 26 and older), the median number of interfaces used independently was 2 (IQR = [1.5, 5.75], maximum = 8).

Group differences in the mean number of interfaces that children could use independently were examined, contrasting based on age group (preschool vs. children vs. teenagers vs. young adults vs, adults), learning disability (with vs. without), language ability (verbal vs. learning/delayed), and reading level (fluent vs. learning vs. non-reader). An ANOVA revealed that there were significant differences between age groups on number of interfaces used independently, *F*(4, 383) = 5.05, *p* < .001. Tukey HSD comparisons revealed significant differences between preschool children (mean = 4.52) and adults (mean = 3.09; *p* = .01), and between children (mean = 5.06) and adults (*p* < .001). No other comparisons were statistically significant. Individuals with a learning disability (mean = 5.4) independently used more devices than individuals without a learning disability (mean = 4.82; *t*(339.53) = − 2.32, *p* = .02). Individuals with phrase speech and above (mean = 5.97) independently used more devices than individuals who were learning to speak (mean = 3.14; *t*(189.95) = 122.3, *p* < .001). Tukey HSD comparisons confirmed significant differences between all three types of readers, showing that fluent readers could use more interfaces than those who were learning to read (*p* < .001), and those learning to read could use more interfaces than those who could not read (*p* < .001).

### Frequently Used Software and Functions

**Fig. 2:**
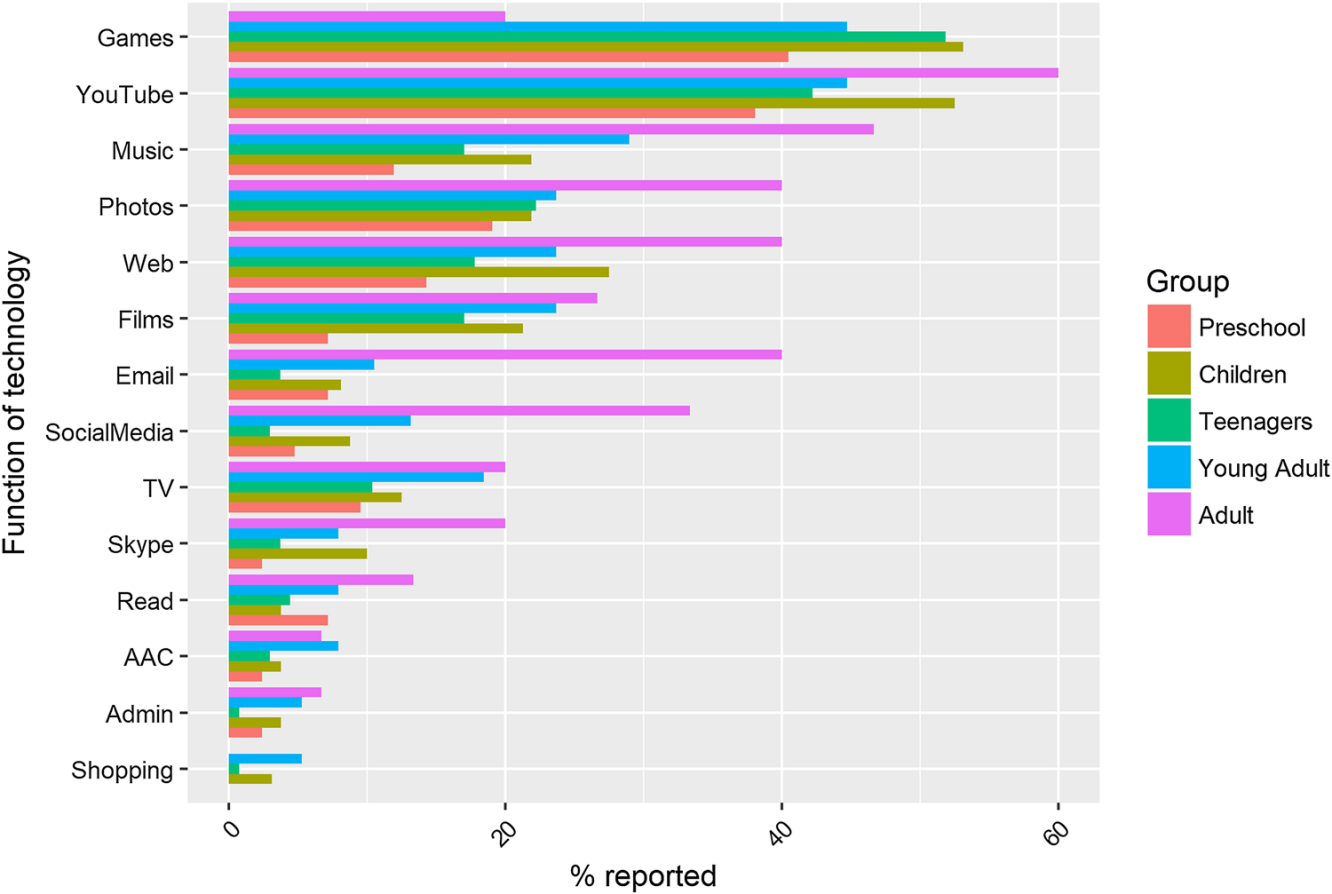
Functions of technology use by children and adults

The ‘function’ of a technology in this context refers to the purpose for which parents report it is used for by their child. A closed-ended set of options were presented in the survey, allowing parents to choose all that applied to their child. Options included reading, playing games, listening to music or browsing the web, etc, as well as an open-ended “other” option. For both children and adults, frequency counts by device-type showed that the most common uses of technology were playing games, watching YouTube and listening to music (see Fig. 2). The least popular uses of technology were shopping, administration, and AAC. There did not appear to be notable differences in the patterns of technology functions by age group.

### Autism-Specific Technology Use

**Fig. 3:**
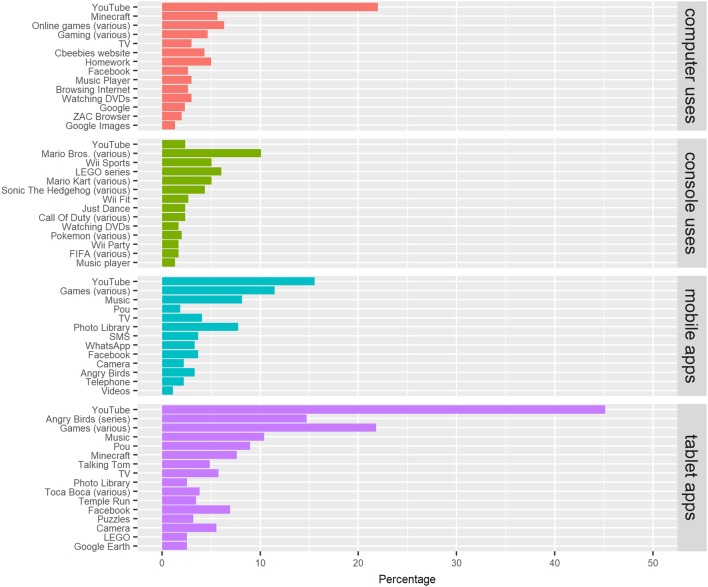
Popular apps and software by device

The top 10 apps most frequently reported by survey respondents in the UK and Spain are presented in Fig. 3. By far, the most popular apps across all devices were YouTube and video/mobile games, plus popular characters or “top-grossing” apps like Angry Birds™, Pou™ (Spain only) and the Toca Boca series. Across all participants and apps mentioned, only one autism-specific app made it into the top ten, and that was reported in Spain only—ZAC Browser™ (https://zacbrowser.com/). Other autism-specific apps were sparingly referenced within the data, and parents who did report use of autism-specific technology were more likely to write “apps for autism” (sic), rather than name specific applications. The pattern of specific apps and types of apps used by autistic people between groups, as well as across countries, appear similar and some of the same popular applications appear across multiple devices (e.g. YouTube™ and Minecraft™).

### Time Spent in Technology-Mediated Activities, Breakdown by Device, and Predictors

In Table 6 we can see that tablets (especially Apple products) are used for longer durations than most other technologies: more than an hour per day on average. For all age groups, the most used devices were iPads (mean across groups = 81.19 min), other tablet brands (mean = 54.89 min), and PCs (mean = 70.61). Gaming devices were reportedly more popular, and used for longer by children, teenagers, and young adults than in other groups.

**Table 6:** Average time spent in technology-mediated activities by device

Mean time* (*n* with access)	Preschool (*n* = 134)	Children (*n* = 160)	Teenagers (*n* = 38)	Young adults (*n* = 14)	Adults (*n* = 42)
iPad	80.8 (68)	80.1 (98)	93.8 (18)	95.4 (11)	65 (18)
Tablet	48.1 (54)	48.1 (55)	67.7 (9)	144 (3)	82.3 (14)
iPhone	21.7 (34)	64.3 (57)	103.8 (13)	54 (5)	37.5 (8)
Smartphone	12.5 (44)	32.2 (61)	40.8 (12)	32 (5)	16 (15)
Blackberry	10 (1)	12.7 (11)	0 (2)	360 (1)	0 (0)
PC	56.2 (91)	80.9 (119)	118.4 (25)	80 (11)	38.3 (24)
Apple Mac	36 (16)	69.6 (28)	18 (16)	2 (3)	24.3 (12)
Wii	17.6 (52)	15.1 (84)	13.1 (16)	60 (3)	3.3 (12)
PlayStation	27.2 (30)	39.8 (44)	30 (8)	44.4 (9)	7.1 (7)
Nintendo DS	11.1 (51)	26.7 (78)	12.1 (14)	13.3 (6)	4.1 (12)
Xbox	32.3 (17)	88.37 (37)	28.5 (7)	25 (2)	60 (6)
Kinect	10 (8)	0 (11)	3.33 (3)	0 (0)	0 (4)

*In *minutes per day*. Note that each estimate draws on a different underlying sample, since different numbers of respondents had access to each device type, and so data have varying levels of reliability

For each participant, the median length of time they were reported to spend using technology per day (across different devices) was calculated. A regression examined the influence of individual age, presence of learning disability, language ability, reading ability, and the number of devices the individual can access on the total time that the individual reportedly spent using technology (see Table 7 for results). The significant predictors of time spent using technology were the individual’s reading level and the number of devices they could access in the home: in both cases higher levels indicated longer time periods. The individual’s age, presence of learning disability, and language level did not predict time spent using technology.

**Table 7:** Predictors of children’s reported time spent using technology

Predictor	*b*	*b* [LL, UL]	*sr* ^2^	*sr*^2^ [LL, UL]
Intercept	81.79	[− 7.34, 170.93]		
Child age	0.98	[− 1.28, 3.24]	0.00	[− 0.01, 0.01]
Child additional needs	15.17	[− 27.65, 57.99]	0.00	[− 0.01, 0.01]
Child language level	− 35.17	[− 97.41, 27.08]	0.00	[− 0.01, 0.05]
Child reading level	− 92.55**	[− 150.56, − 34.53]	0.00	[− 0.01, 0.01]
Number of devices accessed	42.68**	[31.52, 53.84]	0.13	[0.07, 0.19]
Model fit	0.227** [0.14, 0.29]			

*Indicates *p* < .05; ** indicates *p* < .01

A significant *b*-weight indicates the semi-partial correlation is also significant. *sr*^2^ represents the semi-partial correlation squared. Square brackets are used to enclose the lower limits (LL) and upper limits (UL) of a confidence interval

### Parent Attitudes in Relation to Technology Use and Demographics

**Fig. 4:**
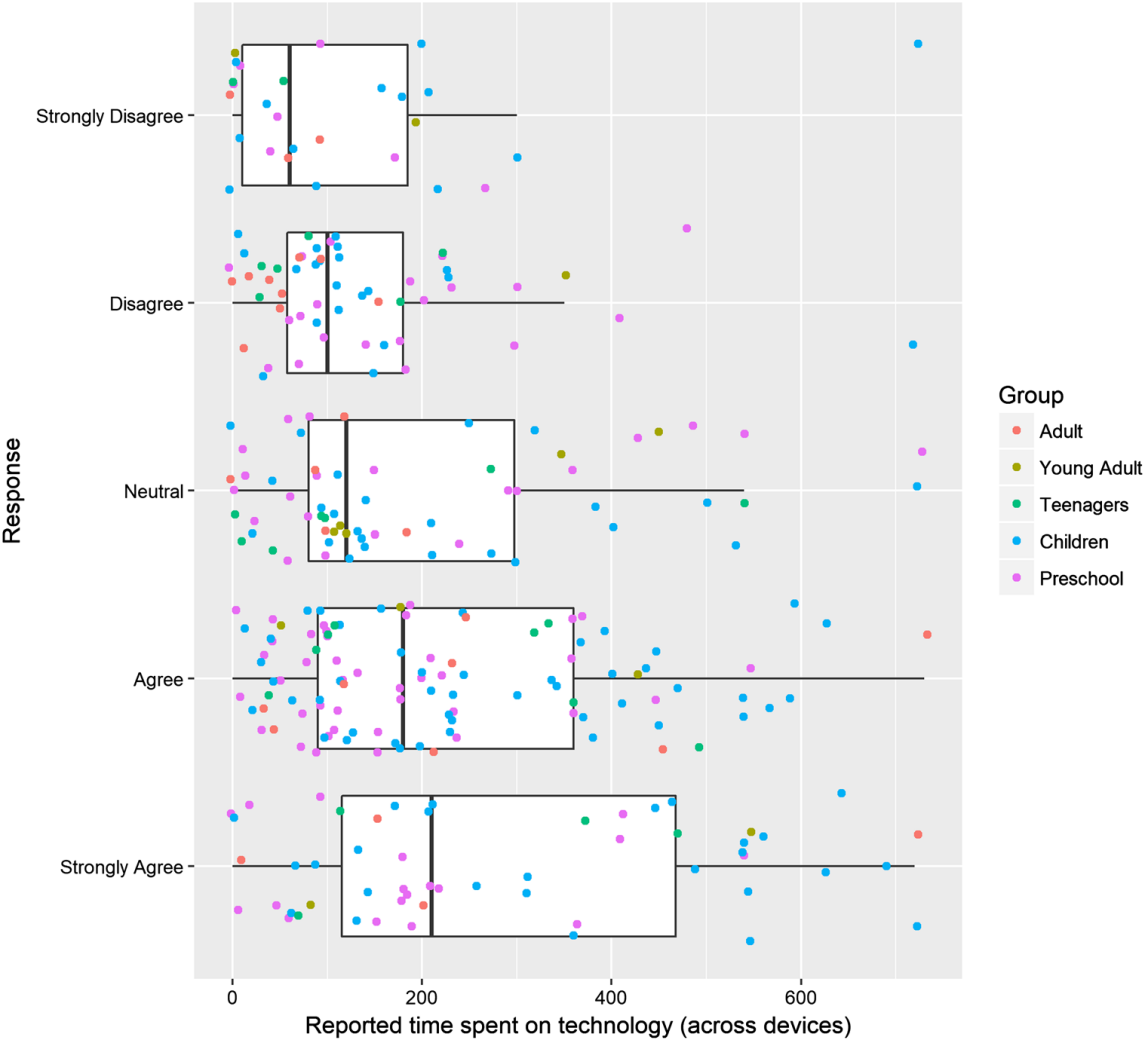
Boxplot on relationship between parent attitude and reported time on child’s technology use

Parents were asked whether they were worried about the time their child spent using technology (scored on a 5-point scale from strongly disagree to strongly agree), and this was compared to total reported time spent using technology (cumulative across devices). An ANOVA revealed a significant relationship between parents’ concern, and the actual time their child spent using technology (*F*(4, 307) = 6.31, *p* < .001). Parents who were more concerned about how much time their child spent on technology reported that their child spent longer using technology than parents who were less concerned (see Fig. 4).

**Fig. 5:**
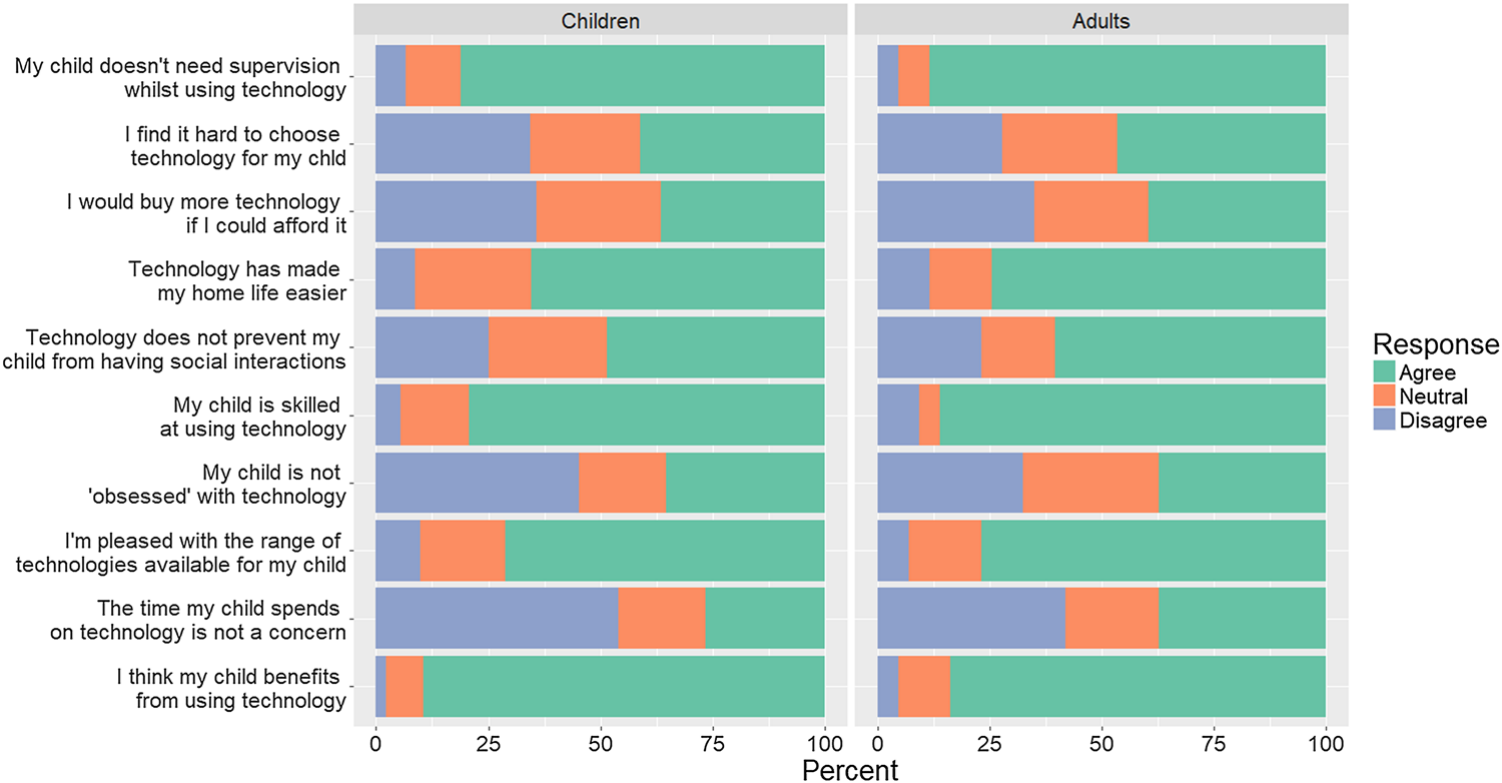
Responses to all items on thoughts about technology

The survey contained ten questions about parents’ thoughts about their child’s technology use (see Fig. 5). Of these, three items (“I worry about how much time my child spends using technology”, “I have had problems with my child being obsessed with technology”, and “Technology prevents my child from interacting with other people”) were summed into a scale capturing attitude to technology, with a Cronbach’s alpha of 0.76, indicating scale reliability. The median attitude score for the whole sample was 9 (IQR = [7, 11], range = 3–15), where 3 = most negative/worried attitude and 15 = most positive/relaxed attitude. An ANOVA reported that attitude score did not differ between age groups of children (*F*(4, 304) = 2.269, *p* = .06).

A regression analysis explored whether parent factors (parents’ age, and age left education) and child factors (age, presence of learning disability, reading and language level, the number of devices they accessed in the home) were related to the reported time that individuals spent using technology (see Table 8). The only significant predictor of parent attitude to technology was the individual’s reading level: better reading was associated with more time spent using technology.

**Table 8:** Linear regression on technology attitudes and parent and child demographic factors

Predictor	*b*	*b* [LL, UL]	*sr* ^2^	*sr*^2^ [LL, UL]
Intercept	8.61**	[5.22, 12]		
Parent age	− 0.03	[− 0.07, 0.01]	0.01	[− 0.01, 0.02]
Parent age left education	0.04	[− 0.03, 0.11]	0.00	[− 0.01, 0.02]
Child age	0.03	[− 0.01, 0.07]	0.01	[− 0.01, 0.03]
Child learning disability	0.23	[− 1.7, 2.16]	0.00	[− 0.00, 0.00]
Child reading level	2.18**	[0.92, 3.45]	0.04	[− 0.00, 0.01]
Child language ability	− 0.07	[− 1.1, 0.97]	0.00	[− 0.00, 0.00]
Child number of devices accessed	− 0.06	[− 0.25, 0.14]	0.00	[− 0.01, 0.01]
Model fit	0.106** [0.02, 0.15]			

*Indicates *p* < .05; **indicates *p* < .01

A significant *b*-weight indicates the semi-partial correlation is also significant. *sr*^2^ represents the semi-partial correlation squared. Square brackets are used to enclose the lower limits (LL) and upper limits (UL) of a confidence interval

## Discussion

This paper asks how children with autism use digital technology in their homes, as reported by parents, and includes data from grown-up offspring. We probed what types of technology, interfaces and software were used, and the time spent on technology-mediated activities, specifically asking whether parents would report high-levels of use of autism-specific technologies. We also explored parent attitudes towards their child’s technology use and potential drivers of variability in these attitudes.

### How Technology Is Being Used by Autistic People

Our data support research that has found that technology is a common recreational activity for autistic people (Fletcher-Watson and Durkin 2015; Mazurek et al. 2012; Mazurek and Wenstrup 2013), extending this work to show that newer mobile and touchscreen technologies are being used by children from very young ages, and by those with a range of language and reading abilities. Individuals with higher reading and language abilities reportedly had access to more devices, more independent usage of different interfaces, and spent more time using technology, than children with lower reading and language abilities.

We found that individuals with a learning disability were reportedly able to use more technology interfaces independently (e.g. mouse, keyboard) than individuals without a learning disability. This was an unexpected result, which does not fit with the remainder of the profile—where individuals with a learning disability showed trends of having access to fewer devices, spent less time using technology, etc. One possible explanation is that parents put effort into seeking a range of different interfaces for their child, when there is difficulty accessing the mainstream interfaces like keyboard and mouse. It is also possible that parents of children with these profiles were more involved in instructing and supporting their child’s use of these interfaces, which may also be reflected in parental reports of skill acquisition. In addition, children were reported as using significantly more interfaces independently compared with adults. It is possible that this reflects the fact that adults are still using the technologies that were available to them as children and have not expanded the range of interfaces that they use so widely. It is also possible that our adult group contains a slightly larger representation of learning disability and other complex needs, since we assume that these autistic adults still live at home. These data further point to the complex interaction between parental attitude, child ability, age and technology use, which requires observational methods and further qualitative analysis to explore.

Reports of technology access in our sample may have been influenced by the fact that it was circulated online, and overtly advertised as a survey about technology and autism. Nonetheless, in this large sample from three countries, drawing on parent-reports about children having a wide range of ages, ability levels and demographic profiles we see evidence that autistic adults and children with autism are competent in using a range of devices and interface-types.

In our sample, parents rarely report use of technologies specifically designed for children with autism, including alternative and augmented communication technologies (AAC). Few parents reported using dedicated AAC hardware, nor AAC-apps on multi-function devices. These data point to a need for continued investigation of barriers to using communication devices with children with autism (Baxter et al. 2012) alongside continued exploration of their effectiveness for improving communication behaviour in children with limited verbal ability (van der Meer and Rispoli 2010). We continue to believe that design specifically for children with autism has strengths (Fletcher-Watson et al. 2016) not least in empowering children through the design process itself (Frauenberger et al. 2013). Nonetheless, our data show how prevalent commercial, ‘mainstream’ technologies are in the lives of autistic people—potentially creating a valuable connection between autistic people and their peers (Ward et al. 2018). Technology is one of the most popular items of ‘special interest’ in the autistic population, and recent research has revealed that these special interests are paramount to well-being in autistic people (Grove et al. 2018). An important future research direction is to create an evidence-base for the technologies which children with autism are currently using. This is no small challenge, since it is often a matter of personal opinion what a ‘positive’ result might be from using any particular technology, and unrealistic to deliver a randomised control trial for each new technology, even in those cases where a clear, beneficial and measurable target outcome can be identified.

Here, the most commonly-reported functions of technology use were digital games, YouTube, listening to music and looking at or taking photos. However, such functions should not be automatically supposed to be mere entertainment. Parents used the open-text sections in the survey to highlight additional and indirect benefits to using technology recreationally (e.g. “she learns to spell by searching for things on YouTube, her vocabulary has increased by millions this summer!”). Other studies have suggested that children with autism may more readily communicate and play together whilst using technology compared to analogue counterparts (Farr et al. 2010; Hetzroni and Tannous 2004). Thus further, in-depth study of our qualitative survey data, and using observational data, is required to determine how our categorical description of usage is manifest in greater detail. Moreover, such work should be conscious of the fact that technology use that seems to be non-functional (e.g. repetitive watching of the same YouTube clip) may have an important role in the life of the individual. Some functions of technology may be relaxing, soothing or provide important cultural knowledge required for social interactions with peers.

### Parental Attitudes to Technology

Parents with concerns about how much time their child used technology did indeed provide higher estimates of this time. However, reliance on parent-report data make this result hard to interpret. It is possible that parents who are concerned about ‘screentime’ inflate reporting of time on devices as a result, or vice versa, that parents with fewer concerns underestimate their child’s time on devices.

The only significant predictor of parents’ attitudes to technology was child’s reading level, and no other factor relating to parent or child were significant predictors. Individuals with higher reading comprehension may access a wider range of online or digital material, and may be more independent in doing so, which could result in parental concern. For example, one parent commented in the survey saying that their child “won’t tell me what he uses the iPad for.” More qualitative research could focus on where these concerns come from (i.e. is there a specific type of technology activity where time is a concern?) and whether these shape parents’ behaviour around their child’s use of technology (Kuo et al. 2015).

### Limitations and Future Directions

This study has some clear limitations, the most important one being that all results rely on parent reports of their child’s technology use. Parental biases may be present in the data, particularly around ‘screentime’, which has been a topical debate in the media recently. Parental bias may also arise from potentially unequal demographic representation in our data—where it seems that a larger portion of our sample from UK and Spain come from lower occupational status. These data are hard to interpret without a deeper analysis of cross-cultural differences in employment and socio-economic status. Nonetheless, across the whole sample we seem to have a fairly good representation of what could be inferred as different socioeconomic and demographic backgrounds, and we make no comparisons between our groups based on country of residence or demographic background.

Furthermore, estimates of time may be confounded by simultaneous use of multiple devices (Smith and Boyles 2012), and therefore these data should be interpreted with caution. Additionally, we cannot make comparisons of technology use across different days of the week. Mazurek and Wenstrup (2013) report that children with and without autism access more technology on a typical weekend than during the week. Nor do we make comparisons on technology use between groups of autistic and non-autistic individuals, though other reports find little difference between these groups, most notably in a large and representative sample as described in Montes (2016).

Our findings about the types and uses of technology by autistic people in the home have a number of implications for design and future research. The first, is that the design of new technologies for autistic people need to be competitive or at least equivalent to currently available technologies and apps, which we now know that autistic people are regularly using. For designers, this may mean designing technological interventions from popular characters such as *The Transporters DVD intervention* by Golan et al. (2010), which was based on the popular *Thomas the Tank Engine* series. Our results also have implications for the evaluations of technology in the everyday lives of autistic people. For instance, a number of studies have reported a mismatch between technologies that have an evidence-base and technologies that are available to autistic people and their families (Kim et al. 2018; Ramdoss et al. 2011), and in this report, we find that these technologies, which are developed for and marketed towards autistic users, are rarely used. One way to move forward would be to build an evidence-base for the technologies which are readily available and being used by autistic people, such as video games (Mazurek et al. 2015) and social media (Ward et al. 2018).

The patterns of technology use reported by parents of children with autism in this study do not seem radically different from what would be expected of a group of children without autism. Technology is most commonly used to play games, listen to music, watch videos, and do homework, and we find that the reported use of autism-specific applications is low. We report that parents do have concerns about children’s use of technology, particularly related to time spent on devices and the social consequences of using technology. Future research could use qualitative and observational methods to elucidate the nature of these concerns.

## Footnotes

1 There are mixed opinions within the autistic community on preferred language, though the trend seems to be towards identity-first language (Kenny et al. 2016). On the other hand, most parents prefer person-first language and we are indebted to them as the participants in this study. Therefore, we will use person-first language to refer to children and identity-first language when talking about adults/autistic people of all ages.
